# Spp24 is associated with endocytic signalling, lipid metabolism, and discrimination of tissue integrity for ‘leaky-gut’ in inflammatory bowel disease

**DOI:** 10.1038/s41598-020-69746-w

**Published:** 2020-07-31

**Authors:** Valerie C. Wasinger, Kenneth Lu, Yunki Y. Yau, Justin Nash, Jess Lee, Jeff Chang, Sudarshan Paramsothy, Nadeem O. Kaakoush, Hazel M. Mitchell, Rupert W. L. Leong

**Affiliations:** 1 Bioanalytical Mass Spectrometry Facility, Mark Wainwright Analytical Centre, The University of New South Wales, Sydney, NSW Australia; 2 School of Medical Sciences, The University of New South Wales, Sydney, NSW Australia; 3 Gastroenterology Department, Concord Repatriation General Hospital, Hospital Rd, Concord, NSW Australia; 4 School of Biotechnology and Biomolecular Sciences, The University of New South Wales, Sydney, NSW Australia; 5 Department of Gastroenterology, Bankstown-Lidcombe Hospital, Eldridge Rd, Bankstown, NSW Australia

**Keywords:** Biochemistry, Biological techniques, Systems biology, Biomarkers, Gastroenterology

## Abstract

Epithelial barrier injury allows contaminants to cross-over into the blood stream and trigger an inflammatory response, contributing to inflammatory bowel disease (IBD). Currently there is no single test that can reliably diagnose intestinal mucosal barrier function or measure impaired epithelial cell integrity associated with increasing permeability. Here, we assess the association between serum proteins and small intestinal permeability as detected by confocal laser endomicroscopy (CLE); in particular the known IBD marker—secreted phosphoprotein 24 (SPP24) and its binding partners; and use developed monoclonal antibodies to assess the role of SPP24 in mucosal healing. Sera were obtained from 28 IBD patients and non-IBD controls undergoing CLE with scores ranging from low to high permeability, as well as active ulcerative colitis from 53 patients undergoing fecal microbiota transplant therapy (FMT). Higher permeability associated with altered lipid metabolism, heightened innate immune response and junctional protein signalling in UC patients. A correlation between increasing leak and SPP24 peptide was observed. There is a strong indication of the novel role of SPP24 in gut barrier dysfunction particularly in ulcerative colitis. Its correlation to the established CLE for monitoring permeability has the potential to provide a blood based parallel to monitor and guide therapy more readily across a broad spectrum of illnesses for which ‘leak’ dominates the pathology.

## Introduction

The capacity to maintain epithelial cell integrity in the gastrointestinal tract (GIT) is crucial for the prevention of chronic intestinal disorders. The ability to re-establish the epithelial barrier following injury is achieved by epithelial restitution followed by wound healing and involves cell proliferation and differentiation. The mature cells lining the GIT include enterocyte, secretory, goblet, enteroendocrine, and dendritic cells; with damaged cells being extruded and replaced by proliferative precursor cells sequestered in intestinal crypts^1^. From a structural perspective, these cells are held together by the interaction of numerous proteins including, tight-, adherens- and gap-junctions (apical-to-basal orientation) in association with intracellular actins and cytoskeletal proteins that reach across the paracellular space providing a physical cellular barrier to the lumen to minimise leak. A mucus layer containing bioactive peptides and hormones provide both a chemical and physical barrier to dietary, toxic and immunogenic compounds and enteric microbiota and pathogens. Barrier dysfunction is defined as a reduction in the continuous layer of epithelial cells and disruption of the junction proteins^2^ that affects the trans/paracellular control of compounds, nutrients and bacteria^3^ allowing for increased permeability or ‘leaky-gut’.

It is unclear whether a compromised barrier leads to: (1) an increase in intestinal epithelial cell permeability and precedes mucosal inflammation^4^; (2) the presence of pathogenic or enterotoxin cues contributing to cellular leak^3^; or (3) systemic inflammation leading to increased permeability, allowing translocation of toxins and pathogens to further exacerbate the immune response^3,5^. In all cases, prolonged assault causes the release of cytokines like Tumor Necrosis Factor (TNF) that contribute to the chronic nature of the disease. A recent study of over 1,000 patients showed no significant genetic associations with increased permeability^6^. As many other proteomic studies have demonstrated, linking genotype with a clinical outcome provides limited data on the complex disease mechanisms when biological and functional changes are ruled at the protein level^7,8^. Tissue protein integrity indicators may be translated into treatments which manage and monitor epithelial barrier function to underpin restitution and recovery, particularly in conditions such as inflammatory bowel disease (IBD). IBD is a relapsing and chronic condition affecting the integrity of the gastrointestinal tract and alimentary canal. There is no cure, with the majority of IBD patients remaining under medical care and management for life to reduce and control inflammation and associated destructive consequences of this inflammation. Differentiating characteristics between the two main forms of IBD centre around disease location and involvement of the mucosa and underlying tissue. Ulcerative colitis (UC) presents as continuous inflammation of the colon and affects mucosa and submucosa, while Crohn’s Disease (CD) can be discontinuous and involve any part of the GIT with transmural damage. With many symptoms shared between UC and CD, subjective measures of generalised disease activity (C-reactive protein, calprotectin) can be misleading, while measures associated with long-term positive outcomes are typically dependent on invasive procedures^7^ not conducive to the recurrent usage required to monitor changes in disease progression. Targeting mucosal healing and restoration of the barrier function over generalised symptoms of inflammation in IBD could be part of treatment management with healing correlating well with reduction of treatment and a decrease in leak^9^. In IBD, active disease is preceded by a loss of barrier function even in macroscopically normal areas of the intestine, demonstrating that permeability can occur much earlier in the pathogenesis of the disease regardless of inflammation^10^. In fact, some studies suggest increased permeability correlates better with symptoms (CDAI, DAI scores) rather than endoscopic activity (CDEIS, SES)^11^. In CD, relapse is preceded by an increase in intestinal permeability^12^, despite macroscopic and histological mucosal healing^13^. Intestinal permeability can be measured by the lactulose/mannitol ratio and reflects the differential absorption of large molecules (via paracellular pathways) and small molecules (via transcellular pathways) respectively in the small bowel. However, neither of these sugars can be used to positively assess colonic permeability and are therefore limited in UC or other diseases localised in the colon due to the influence of metabolising microbiota^2^. Other sugars, organometallic chelates, or polyethylene glycol as well as histological and immunohistochemical methods are also used to determine permeability^2^. Invalidation of permeability testing can occur in active inflammation due to the presence of ulcers. Therefore, the modulation of gut-derived microbial factors in the circulation is considered definitive evidence of increased permeability^14^. In recent years, confocal laser endomicroscopy (CLE) has been applied to the study of epithelial barrier function of IBD patients, with restoration of barrier function (as assessed by CLE) representing a state of ‘deep’ remission. This test allows for identification and scoring of inflammation in conditions with distinctive microscopic features such as IBD and is hailed as a potential new ‘gold standard’ of mucosal healing^15^. It requires the use of fluorescein as the contrasting agent and can be used to assess: mucosal epithelial cell drop-out; cell-junction enhancement; and fluorescein loss, to score leak^16^. In a previous study investigating IBD with ongoing bowel symptoms, we have shown that a deficit in permeability and CLS score > 13.1 is associated with irritable bowel syndrome (IBS) like symptoms with 95.2% sensitivity and 97.6% specificity^13^. Permeability or leak has been linked to a wide variety of diseases and the use of CLE is an established application for diagnosis of Barrett's oesophagus, gastric intestinal metaplasia, coeliac disease, microscopic colitis and cancerous polyps^17^. There are no adequate blood-based markers to reduce the need for endoscopy and or confocal analysis.

The SPP24 protein is unique to vertebrates and is made up of three major domains. Of interest is the cystatin-like domain for which two peptides have already been associated with active IBD^18, 19^. This region has been shown to exhibit similarity to the Bone Morphogenic Protein/Transforming Growth Factor-β type II receptor (TRH1 domain)^20^. In addition to bone morphogenic activities as shown by Sun et al.^21^; our data suggests that this protein is modulated in IBD patients^18^, and also has a role in permeability and gastrointestinal epithelial cell integrity. In this study we determine the binding partners of SPP24 and quantitate their ability to differentiate IBD cases of CLE scored variable intestinal permeability in particular UC patients. We demonstrate that an antibody raised against the specific SPP24 peptide epitope is capable of differentiating CLE scored patients; and we apply the SPP24 specific antibody to demonstrate the levels of pre and post treatment SPP24 are significantly modulated in patients responsive to FMT treatment.

## Results

### Higher permeability associates with altered lipid metabolism, heightened innate immune response and junctional protein signalling

**Figure 1:**
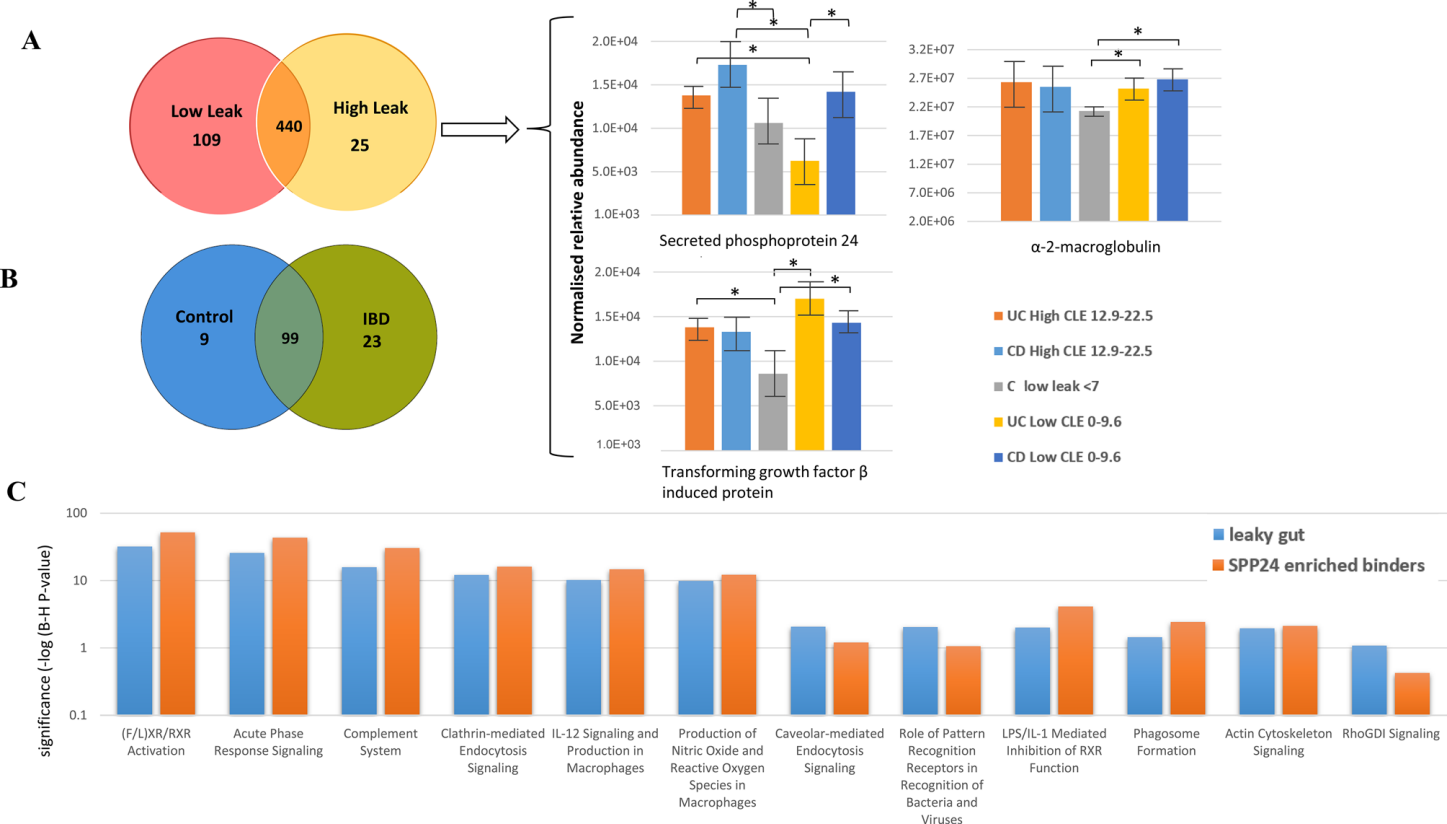
The number of proteins identified based on 2 peptide identification, < 1% FDR significantly changing are shown for (**A**) high leak (CLS score 12.9–22.5) compared to low leak (CLS score 0–9.6) patients. Within this dataset a closer look at subjects with increased CLS scores demonstrated heightened levels of SPP24, α-2-macroglobulin and TGF-β induced protein. Significance was assessed using T-test. Control patients with physiological leak (< 7) showed reduced levels. (**B**) Significant SPP24 binding partners of IBD and control patients. (**C**) Comparative analysis of significantly enriched pathways from leak patients low leak/high leak (blue), and SPP24 binding partners based on EmPAI abundance (orange) demonstrates commonality across FXR/RXR/LXR activation, endocytosis signalling and LPS response amongst the significantly represented pathways. Significance of groups is assessed by p value corrected for multiple testing using the Benjamini-Hochberg (B-H) false discovery rate. Significance p < 0.05 is indicated by dotted line.

The protein abundance variation was assessed using serum collected from 26 patients with recorded CLE scores including: 10 patients with high to moderate leak (CLE = 12.9–22.5), 10 patients with low leak (CLE = 0–9.6), and 6 non-IBD controls (CLE = 0–12.9). Patient details are defined in Table 1 and further details are given elsewhere^13^. Modulations were explored at the protein level using shot-gun proteomic techniques and show differences in the 574 proteins analysed between high and low leak patients (Fig. 1A), and 131 proteins binding to SPP24 analysed between IBD and control patients (Fig. 1B). A closer look at proteins already identified as having an association with SPP24, α-2-macroglobulin and TGF-β were also identified within these datasets^18,22^ and validate the capture approach used here. Significant differences between high and low leak patients with IBD were found to associate to the nuclear receptors important in the regulation of cholesterol and fatty acids called liver-x-receptor (LXR), farnesoid-x-receptor (FXR) and retinoid-x-receptor (RXR). In addition, acute phase response and complement pathways were also found to be significantly altered across both datasets (Fig. 1C). Altered pathways in higher permeability patients also correlated well with the role observed for SPP24 binding partners between IBD and control patients (Fig. 1C orange). A comparison of UC and CD patients demonstrated differences at the pathway level for endocytosis, secreted and signalling pathways including remodelling of epithelial adherens junctions and tight as well as cell-to-cell junction signalling (Table 2). Pathways involving endocytosis, diapedesis and extravasation are particularly enriched in patients with CLS scores above 12.9; and acute phase response and response to external stimuli, lipid binding and response to wound healing are also enriched (Supplementary Table 1). Binding partners also showed association to gastrointestinal disease, organismal injury, cancer, cell-to-cell signalling and a relationship to tissue morphology as the top networks and pathways associated with the binding of proteins to SPP24.

**Table 1:** Subject details and disease characteristics were obtained at time of recruitment and are summarised.

CLS study	Cases	UC (CD)	Mean CLS	CRP mean (Range)	Mean ESR (mm/h)
Low leak	10	5 (5)	4.63	8.79 (0.3–35.4)	19.0
Moderate leak	7	3 (4)	10.79	3.54 (0.3–15.9)	16.7
High leak	5	3 (2)	17.34	22.66 (0.4–23.7)	27.7
Control	6	–	7.54	3.35 (0.4–15.9)	3.9

Active UC FMT study	Cases	Endoscopic remission	Clinical remission		
FMT treatment	31	6	18		
Placebo	22	1	1		

Further patient details regarding these studies are available^13,45^.

**Table 2:**
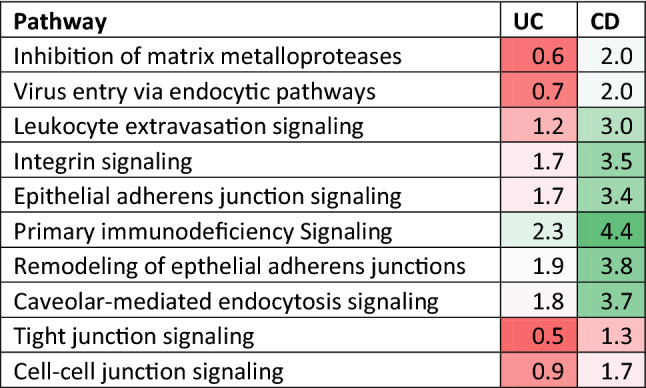
Comparison of UC and CD high leak (CLS > 12.9) pathways significantly perturbed with z-scores > 2 in any group and with >2 fold difference between z-scores.

Colours give a relative indication of change in the pathways (green—increase, red—decrease).

**Figure 2:**
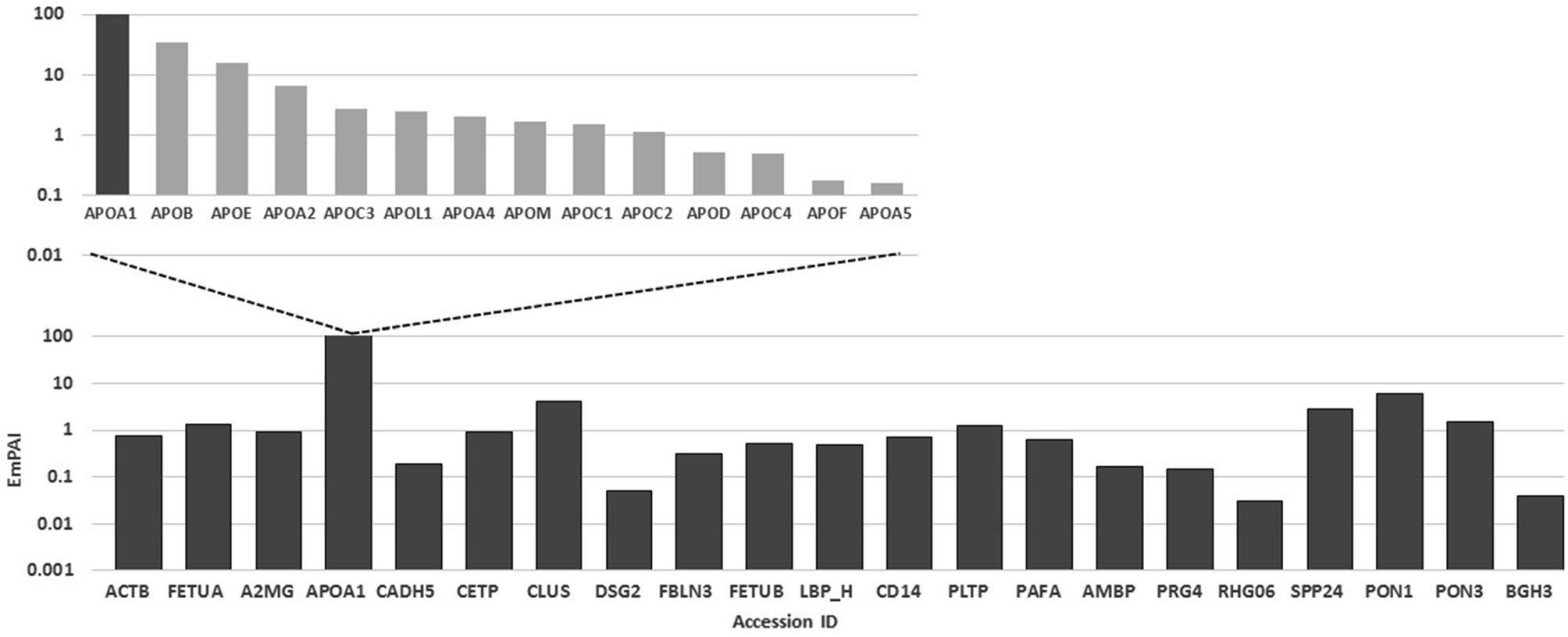
Relative abundance (EmPAI) of primary binding proteins bound to 12 amino acid length peptide VSAQQVQGVHAR. Insert panel shows the Apolipoproteins found to interact with SPP24 peptide.

Identified SPP24 binding partners include proteins already known to interact with SPP24, and also observed in the proteomic comparison of leak patients, such as α-2-macroglobulin, TGF-β (inset Fig. 1A). Non-specific binding partners were reduced by incubating the SPP24 bound proteins with trypsin overnight (prior to elution) to remove enzyme exposed proteins while retaining the directly bound peptides to the SPP24 peptide of interest. These proteins have been given the name ‘primary’ binders. A list of SPP24 peptide primary binders is given in Supplementary Table 2, with a graph of proteins of interest plotted against an abundance indicator-EmPAI shown in Fig. 2. Many of these proteins are linked to the metabolism of lipids. Paraoxonase 1 (PON1) was confirmed to be a direct binding partner of SPP24 in a reverse binding experiment using PON1 antibody as the bait and demonstrating the capture of SPP24 (data provided in Supplementary Table 3).

Previous work identified an increase in SPP24 protein was able to discriminate between IBD and control patients and increasing amounts of SPP24 peptide correlated with an increase in the severity of symptoms- separating those in clinical remission^18^. Here, we are able to show increased levels of SPP24 peptide in UC higher leak patients relative to lower leak patients (Fig. 1A).

### UC Patients entering remission show reduced levels of SPP24 peptide

**Figure 3:**
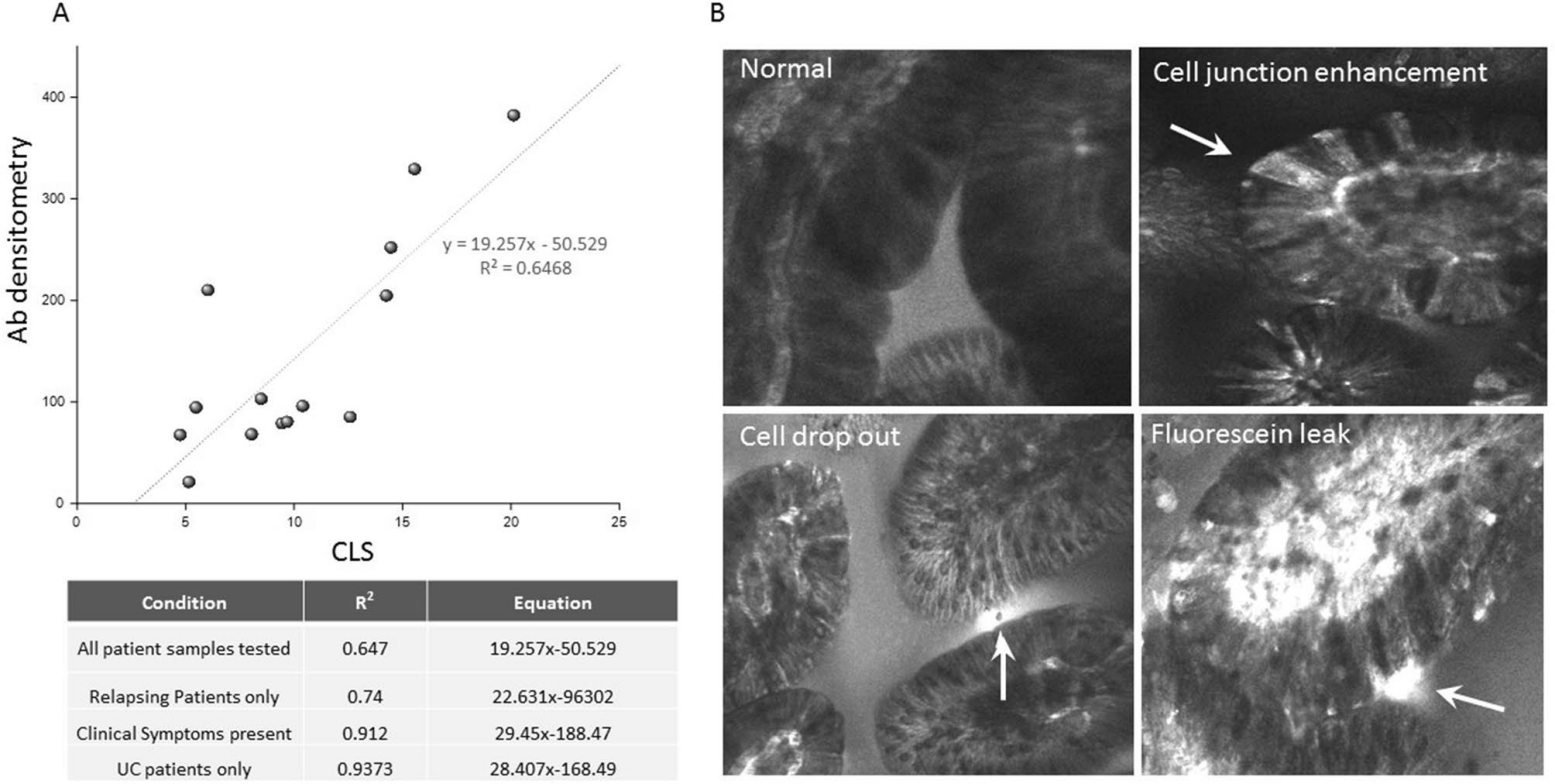
Patients with corresponding CLS and medical histories were assessed for levels of SPP24. (**A**) The graph shows an increase in the amounts of SPP24 present with increasing leak score (CLS). The table summarises the correlation tested for: relapsing patients (n = 8), patients with recorded clinical symptoms (n = 4), and UC only patients (n = 6). The Pearson correlation between the CLS score (barrier dysfunction) and the amount of SPP24 in blood is 0.684. The estimated change in SPP24 per unit change in CLS (the slope), is 0.10 with a standard error of 0.03. The significance level of this t-test is 0.007. (**B**) Confocal endomicroscopic images of terminal ileum showing examples of healthy, cell junction enhancement, cell drop out and fluorescein leak.

Barrier dysfunction as a measure of cell junction enhancement, drop out, and fluorescein leak (CLS leak score) allows for the interpretation of leak as a continuum of severity. It can identify physiological leak in non-IBD patient controls, reduction in CLE score with treatment, and prognosticates clinical (relapse free) remission which is correlated with a low leak score^13^. Here, we can show significance of increased mean amounts of SPP24 in relapsing UC patients with respect to those patients remaining in clinical remission (> 340 days to 2000 days follow up) (Fig. 3). A positive correlation (score = 0.68) was observed between SPP24 peptide amounts and increasing CLE score determined from 14 patients (p = 0.007), with a unit change in SPP24 to CLE of 0.1, demonstrated in Fig. 3 with examples of confocal images of barrier dysfunction.

**Figure 4:**
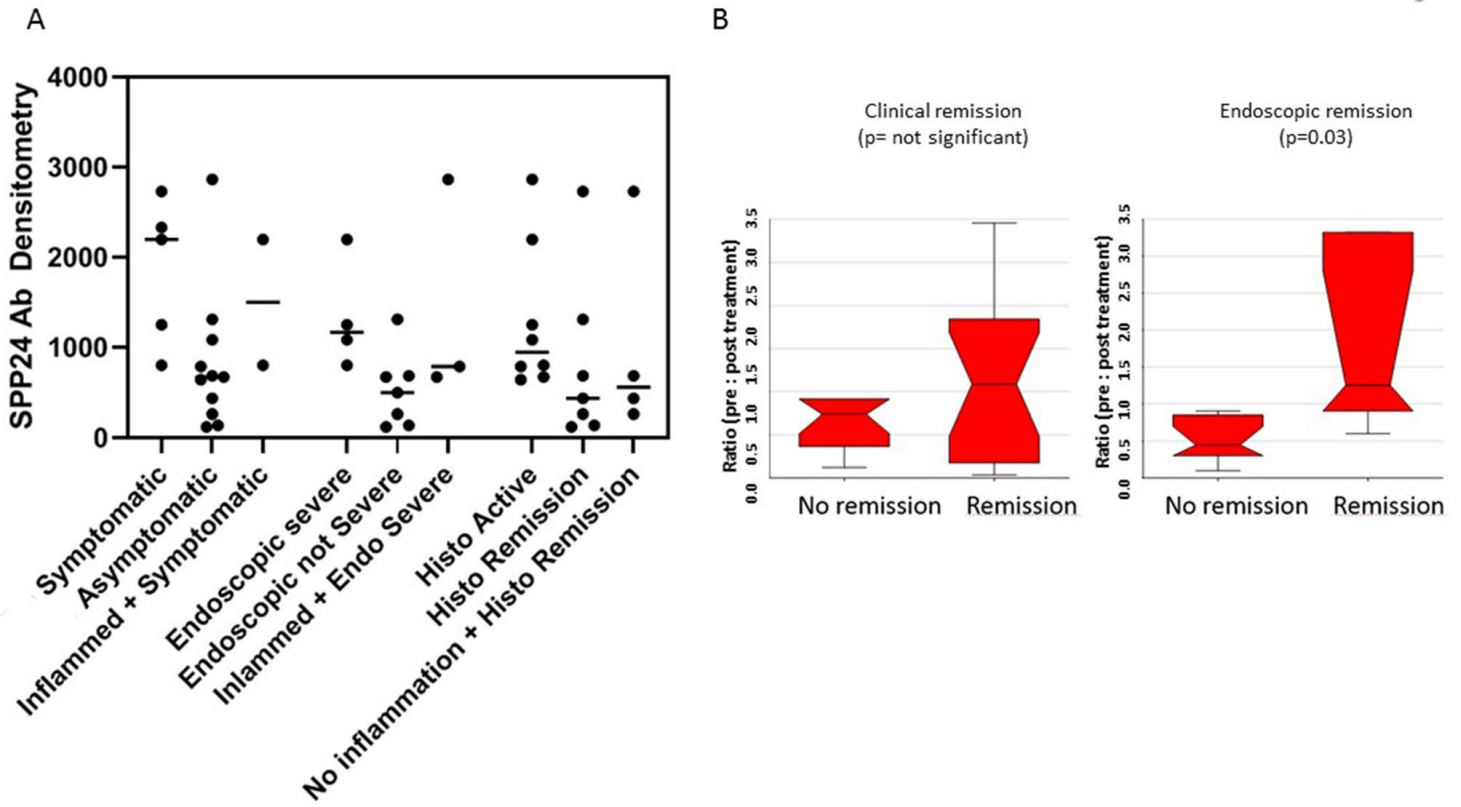
Increased Antibody levels of SPP24 specific peptide in: (**A**) symptomatic, severe and active UC patients, and (**B**) antibody detected levels of the marker SPP24 in UC patients undergoing FMT with the primary endpoint being either endoscopic or clinical remission comparing pre- to post-treatment patients. Significance was assessed using T-test.

Development of monoclonal antibodies raised against the portion of the protein containing the peptide epitope have similarly demonstrated findings from MRM based assays. Antibodies were applied to the CLE patients and demonstrated increased levels of the SPP24 peptide in symptomatic IBD patients compared to asymptomatic patients (Symptoms related to disease activity and bowel movements), and endoscopically severe compared to non-severe patients (indicative of mucosal healing) Fig. 4A. A similar trend to decreased SPP24 peptide was observed when comparing patients categorised as in remission by histological means with inflammation influential (but not the only factor) in the levels of the peptide. These results were similar in a separate study of 53 active UC patients receiving FMT treatment and follow up at 8 weeks post treatment. Clinical remission is an indication of reduction of symptoms, whereas endoscopic remission is dictated by reduced or no visual inflammation during a scope. Using the primary endpoints of clinical and endoscopic remission, 18 patients benefiting from FMT therapy showed a reduction in the level of SPP24 specific peptide post-treatment compared to pre-treatment levels (p = 0.03; Fig. 4B) while non-responsive patients showed no significant modulation of SPP24 peptide pre- to post-treatment.

## Discussion

The exact molecular mechanisms that associate poor intestinal barrier function and increased permeability with IBD are not well understood. This is particularly true with respect to the relationship between permeability, active disease, persistent TNF-α and the presence or absence of symptoms despite macroscopic healing. Remission in the presence of ongoing symptoms not attributable to known inflammatory activity occurs in approximately 39% of IBD cases^23^. Many effectors contribute to increasing permeability and the consequential destabilisation of the cellular structure that ultimately results in chronic inflammation as well as symptoms such as diarrhoea. Studies have implicated junction proteins, intracellular actins, mucins, opportunistic/pathogenic microbiota, toxins, TNF-α, and other factors with altered levels in IBD patients^14^.

The effects of increasing permeability can be measured by confocal leak score in association with activity scores such as the CDAI or Mayo score which considers permeability as well as symptoms. Some insight into the order of events and the mechanics of each contribution can be gained from proteomic studies of stratified confocal leak scored patients. Here, we applied proteomics to determine proteins and pathways associating with increased CLS to address the global effect of all these differential features with respect to barrier dysfunction and permeability in IBD. Serum protein profiles associating with cellular integrity can define the biological pathways contributing to dysfunction and moves the focus from managing generalised symptoms in common to both UC and CD, to bridging the underlying chronic triggers of those symptoms. In addition to a proteomic study on CLS patients, we have used the SPP24 peptide as a bait to capture binding proteins and identify the association with intestinal permeability within the context of IBD. The SPP24 protein has been shown to form aggregates with other proteins^22^, here we can show that the 12 amino acid peptide also has this capacity. The importance of the regulation the Retinoid and Farnesoid nuclear receptors, the role of endotoxin and LPS mediated recognition of microbiota, and lipid metabolism were established in patients with increased permeability using mass spectrometry techniques. Antibodies were generated and employed to assess the levels of SPP24 in confocal leak scored patients and a cohort of UC patients undergoing FMT treatment. The link to endoscopic remission in the epithelial gut lining with a reduction in SPP24 peptide, in addition to the possible role of the microbiome was further established in these patients. Altered permeability in control subjects was observed in this study with modulation of SPP24 abundance. The reason for this permeability was not followed up at the time samples were taken and therefore further insight into the role of SPP24 and permeability in non-IBD conditions needs to be explored in a larger cohort of patients.

Enriched pathway analysis has correlated higher leak with dysregulation of FXR/RXR as the most significantly altered pathway in leaky gut patients. A biochemical link between an altered microbiome and nuclear receptor signalling, alteration in lipid metabolism, fatty acid and bile acid metabolism and lipid oxidation has been noted previously^24,25^. Alterations in IBD high and low density lipoproteins (HDL and LDL) and lipid metabolism are also noted in many studies^26,27^. There is strong evidence for the activation of FXR in the intestinal tract to reduce pro-inflammatory cytokines and therefore limit inflammation and epithelial permeability; providing an entero-protective role through induction of gene transcription^28^. Reabsorption of bile acids can occur in a tightly controlled negative feedback mechanism involving active transporters as well as passive diffusion^24^. Significant differences in high leak patients seen in this study supports the loss of this entero-protection resulting in increased permeability. The demonstrated importance of this pathway in SPP24 binding partners suggest the modulation of RXR and FXR bile acid metabolism and synthesis is regulated by the recognition of endotoxin and LPS, a major component of the outer membrane of Gram-negative bacteria in an IL-1 mediated manner involving CD14/TLR4 at the cell surface and production of proteins involved in the regulation of lipid metabolism and transport. Thus, the role of the microbiome with respect to levels of SPP24 in FMT treated patients was explored further and demonstrated reduced levels of SPP24 protein for endoscopic remission of successful FMT treatment. These FXR/RXR nuclear receptors are also responsible for redox balance in cells affecting gluconeogenesis and resulting in dysregulation of lipid metabolism, oxidative stress, apoptosis, and the release of pro-inflammatory cytokines^29^. Alongside the modulation of RXR signalling pathways, disturbance of pattern recognition receptors (PRR), reactive oxygen species (ROS), and Lipopolysaccharide (LPS) pathways are also indicative of mucosal intestinal barrier dysregulation in IBD patients of epithelial cell permeability. The mirrored pathways in the SPP24 binding partner studies of the CLS patients indicating that SPP24 is likely to be a central component to the deregulated process.

The increase in ROS in inflammatory processes has been linked to reduced levels of antioxidant species particularly in the gastrointestinal tract^30^. Along with a number of peroxidases identified as binding partners to SPP24, of interest are the paraoxonases (PON). The PON enzyme family (PON1, 2, and 3) are antioxidants that are able to degrade oxidised phospholipids^31^, and which play a protective role by binding to HDL. PON1 and PON3 are intimately linked with intestinal barrier dysfunction, and increasing epithelial cell permeability in conjunction with SPP24. These data are consistent with other findings that PON1 is reduced with a concomitant increase in TNF-α and ROS in the colon of DSS-treated mice^30^, and associated with dysfunctional HDL proteins^32^. In combination with Cholesterol Ester Transfer Protein, the lipid binding proteins, and Apolipoproteins significant modulations in lipid binding and metabolism are established. Better regulation of the levels of PON1 in serum of patients with increased epithelial permeability may have relevance in a clinical setting and ameliorate ROS tissue damage and phospholipid turnover, while reducing permeability.

The small GTPases such as RHG06, (involved in cell clearance), Cadherin-5, and the junctional protein DSG2, may indicate an SPP24 relationship with endosome formation, and may be directing cargo towards endosomes rather than secretory pathways of exocytosis^33^. This idea is supported by the comparative analysis of SPP24 binders and CLS scored patients showing modulation of similar pathways of endocytotic signalling. Indeed, quantitative experiments on plasma exosomes failed to detect any SPP24 or PON1 associated with exocytosis in plasma of CLS scored IBD patients (data not presented here).

Opportunistic pathogens have evolved specialist defence evasion capabilities that cover: molecular mimicry (e.g. Sta toxin^34^); convergence of human signalling molecules and microbial metabolites acting on common receptors^35^; Type 3 secretion system that manipulate host signalling pathways^36^ and deliberate engulfment strategies to gain entry^37^. Adhering to the cell surface and the consequential invagination of the membrane to engulf the bacterium is another subversive strategy that triggers an inflammatory response^38^. The invagination of the junctional proteins has been associated with the disruption of the peri-junctional actinomyosin ring and heightened loss of barrier function^39^. Proteomic comparison of CLS scored permeability patients also pointed to enriched pathways of actin signalling. Junctional protein interaction with the actin cytoskeleton is an important mechanism regulating the integrity of the epithelial barrier and controlling paracellular transport. Co-endocytosis of apposed cell membranes containing Claudin-3 following dissociation from ZO-1, Occludin and JAM tight junction components has been observed previously^39^. The immune modulator of both pathogenic triggered barrier dysfunction as well as the symptoms of diarrhoea is TNF^40^, and these morphological changes are influenced by LPS and can cause bacterial translocation, degranulation of mast cells, and serine protease involvement. LPS triggers TNF-α release via LPS binding protein and CD14, which presents the LPS to Toll-Like receptor (TLR) 4^41^. These observations are also reflected in the molecular cross-talk between the microbiome and the host, through the activation of the innate immune system. Activation of the acute phase response is shown to be of significance in both the leaky-gut and proteins associating to SPP24. This relationship between microbiome and host have been noted as key mediators of barrier dysfunction^3^, and therefore may have relevance to FMT therapies.

The binding partners of SPP24 consist of many effector proteins, growth factors and cytokines such as TGF-β and have been implicated in epithelial cell restitution. Signalling between regulatory peptides acting via TGF-β dependent pathways contribute to epithelial cell restitution from the basolateral side and includes cell proliferation, differentiation, apoptosis and migration^42^. While TGF- β independent pathways are associated with goblet cells, and mucin from the apical side of the intestinal epithelium^43^, recent studies show SPP24 down regulates TGF-β with significant implications for bone growth^44^. Numerous N-terminally preserved forms of SPP24 retain biological function; here we have shown the ‘VSAQQVQGVHAR’ peptide is the binding site for proteins involved in epithelial cell integrity. Signalling cross-talk between pathways would provide a reasonable link between the pathways delineated by the diverse SPP24 binding partners.

Epithelial cell permeability as well as mucosal healing is the target in therapeutic care and management of IBD. Mucosal healing is related to a Mayo score of < 1 for UC and SES-CD score < 3 for CD, while CLS is a good indicator of permeability. Serum levels of SPP24 have the potential to be used in clinical applications as a blood-based indicator of permeability as well as an indicator of clinical deterioration requiring treatment escalation. Increasing amounts of serum SPP24 correlated well with increasing permeability (r = 0.684, p = 0.007); and this was also confirmed through the use of specific monoclonal antibodies raised against the peptide epitope in the same CLS scored patients as well as an additional cohort of FMT treated UC patients. FMT is a novel means of manipulating the microbiome, particularly relevant as the intestinal microbiota is implicated in the pathogenesis of UC^45^. Among the 53 FMT treated patients assayed in this study followed at 0 weeks and follow-up at 8 weeks post treatment, 6 patients reached the primary endpoint of endoscopic remission, indicative of mucosal healing. These patients showed a significant trend of a reduction in the levels of SPP24 at the primary endpoint (i.e. pre- to post-treatment ratio increased in remission).

## Conclusion

We have interpreted these results as a strong indication of the novel role of SPP24 in gut barrier dysfunction through modulation in endocytic signalling and lipid metabolism. It correlates to the established CLS, potentially providing a blood based parallel and ability to monitor more readily and to guide therapy. SPP24 in association with its binding partners, specifically PON1, may prove to be useful as both indicators of permeability, barrier dysfunction as well as novel therapeutic targets across a broad spectrum of illnesses for which ‘leak’ dominates the pathology.

## Materials and methods

### Experimental design and rationale

IBD and control patients were recruited from IBD ambulatory clinics of Bankstown-Lidcombe Hospital and Concord Repatriation Hospital as described previously^18^. Patients were aged between 18–70 years old and provided written informed consent. Predetermined exclusion criteria included known-IBS, celiac disease, intestinal resection surgery (apart from limited ileal resection for CD), pregnancy or breast-feeding, renal disease, diabetes mellitus, decompensated liver disease, regular use of non-steroidal anti-inflammatory drugs (NSAID) or known allergy to fluorescein. Control patients included those found to have no gastrointestinal disease and/or those undergoing endoscopy with normal findings. IBD diagnoses were confirmed by histological and endoscopic criteria with at least 12 months duration. All subjects had their phenotype confirmed by a gastroenterologist with radiologic and/or endoscopic evidence within 30 days from blood sampling as part of their routine care. Disease-specific activity indices for CD and UC; Crohn’s Disease Activity Index (CDAI), UC Partial Mayo Score (PMS), with paired biochemical markers of inflammation, c-reactive protein (CRP) and erythrocyte sedimentation rate (ESR) were collected.

Study one was a prospective study of 28 age and sex matched patients with recorded confocal endomicroscopy (CEM) using fluorescein contrast to detect three features representative of cell shedding in the intestinal mucosa^16^ scores measured. Briefly, the three features that were identified included: (1) cell junction enhancement (CJE); (2) cell drop out or shedding (CDO); 3) fluorescein leak (FL). A composite score measuring the severity of ‘leak’ in the gut as a continuous variable was designed that combines FL, CJE and CDO. The range is from 0 indicating complete absence of leaky gut to > 20, indicating an increased severity of leak and leaky gut. In this study, 10 patients with moderate to high leak and IBD (scores = 12.9–22.5), 10 patients with low leak (scores = 0–7.5); and 6 non-IBD control patients (scores = 0–12.9) were assessed. Subject details and disease characteristics were obtained at time of recruitment and are summarised in Table 1.

Study two involved active UC patients undergoing either colonoscopic FMT infusions (n = 31) or placebo (n = 22) infusions via the lower GIT with plasma collection at week 0 and 8. The primary endpoint at week 8 was clinical remission and endoscopic remission or response at week 8. We focused on patients which achieved clinical remission (n = 18), or endoscopic remission (n = 6) indicative of mucosal healing, to assess levels of SPP24 peptide pre and post treatment compared to non-responsive patients (n = 13).

Clinical data collected from IBD subjects included disease phenotype, extent of disease, duration of disease, smoking status and NSAID-use. Symptoms prior to the CLE bowel preparation were recorded in a diary. The Crohn's Disease Activity Index (CDAI, symptomatic defined as ≥ 150) was used for CD and partial Mayo score (symptomatic defined as ≥ 2) for UC. Serum inflammatory markers of erythrocyte sedimentation rate (ESR, upper normal limit 16 mm/h) and CRP (upper normal limit 6 mg/L) were recorded. Endoscopic activity was measured by the Crohn's Disease Endoscopic Index of Severity (CDEIS) for CD and the Mayo endoscopic sub-score for UC. Mucosal healing was defined as a CDEIS of 0 for CD or Mayo endoscopic sub-score of 0–1 for UC. Histological biopsies also had to demonstrate quiescent disease for those meeting the definition of mucosal healing, which was defined as absence of epithelial breach (ulceration, erosions) and inflammation (cryptitis, crypt abscess, neutrophilic infiltration). Non-acute inflammatory changes (crypt distortion, branching) were not excluded. Fecal culture was performed to exclude infection where relevant^13,45^.

### Collection and Storage of serum

Patient blood samples were collected using standard venepuncture techniques for serum (gold-top) and centrifuged at 4,000 rpm for 10 min at room temperature. Serum was extracted and separated into 100 μl aliquots and stored at −80 °C.

### Bait and capture of SPP24

Tosylactivated magnetic beads (*Thermo Scientific,* Illinois, USA) were used as per manufacturer’s instructions. Synthetic SPP24 peptide, was bound to the magnetic beads. Pooled whole serum samples grouped into UC (5 patients), CD (5 patients) and controls (5 patients) were analysed for binding partners. Primary binding partners were determined by washing the captured serum bound proteins (PBS pH 7.4, 0.1% Tween20, followed by PBS pH 7.4) prior to incubation overnight at room temperature with trypsin 10 µg. This was followed by repeatedly washing the captured proteins still bound after digestion. The primary binding partners were then released using 0.15% TFA and also underwent analysis by LC–MS/MS. Reverse binding studies were also carried out using polyclonal PON1, CD14 (PA-29588, PA5-28997; Thermofisher), NCKPA1 (NBP1-83269; NovusBio) to confirm the relationship. Eluted proteins underwent trypsin digestion and proteins were analysed by ESI-LC–MS/MS using an Orbi-trap MS instrument (Thermo Electron, Bremen, Germany).

### Mass spectrometry

Following previously described methods^46^, all samples were concentrated with C18 stage tips *(*Thermo Scientific, Illinois, USA*)* according to the manufacturer’s recommendations except that the elution buffer consisted of 80% CH_3_CN, 0.1% Formic acid. Digested peptides were reconstituted in 10 μL 0.1% formic acid and separated by nano-LC using an Ultimate 3,000 HPLC and autosampler (Dionex, Amsterdam, Netherlands). The sample (1.0 μL, 10% of sample) was loaded onto a micro C18 pre-column (300 μm × 5 mm, Dionex, Scoresby, VIC, Australia) with Buffer A (98% H_2_O, 2% CH_3_CN, 0.1% TFA) at 10 μL/min. After washing, the pre-column was switched (Valco 10 port valve, Dionex) into line with a fritless nano column (75 μm i.d. × 15 cm) containing reverse phase C18 media (1.9 μm, 200 Å Dr Maisch GmbH). Peptides were eluted using a linear gradient of Buffer A to Buffer B (98% CH_3_CH, 2% H_2_O, 0.1% formic acid) at 0.25 μL/ min over 60 min. High voltage (2000 V) was applied to low volume tee (Upchurch Scientific, Oak Harbor, WA, USA) and the column tip positioned 0.5 cm from the heated capillary (*T* = 280 °C) of an Orbitrap Velos (Thermo Electron, Bremen, Germany) mass spectrometer. Positive ions were generated by electrospray and the Orbitrap was operated in a data-dependent acquisition (DDA) mode. A survey scan 350–1,750 m/z was acquired in the Orbitrap (Resolution = 30,000 at 400 m/z, with an accumulation target value of 100,000 ions) with lockmass enabled. Up to the 10 most abundant ions (>5,000 counts) with charge states + 2 to + 4 were sequentially isolated and fragmented within the linear ion trap using collisionally induced dissociation with an activation *q* = 0.25 and activation time of 30 ms at a target value of 30,000 ions. The m/z ratios selected for MS/MS were dynamically excluded for 45 s.

### Protein identification

MS ion abundance was analysed using ProgenesisQI for proteomics v2.4 (Nonlinear Dynamics, Newcastle upon Tyne, UK). Ion intensity maps from each run were aligned to a reference sample and ion feature matching was achieved by aligning consistent ion m/z and retention times, normalized against total intensity (sample specific log-scale abundance ratio scaling factor), and compared between groups by one-way analysis of variance (ANOVA, p ≤ 0.05 for statistical significance) as previously described^18^. Type 1 errors were controlled for by False Discovery Rate (FDR) with q value significance set at < 0.01. Non-redundant NCBI database (downloaded 29 January 2015 containing 57,851,050 sequence entries) was searched using Mascot Daemon (Matrix Science, London, England) with the following parameters: 4 ppm peptide tolerance and 0.4 Da fragment tolerance, ‘All-species’ and ‘semi-tryptic’, and variable modifications to: Methionine (Oxidation); Serine, Threonine, Tyrosine (Phosphorylation), selected to generate peak lists. Only peptides with an ion score > 20 were considered for protein identification. Enrichment pathway analysis was achieved using Ingenuity software (Qiagen, Limburg, Netherlands) using the proteins listed in Supplementary Table 2.

### Anti-SPP24 peptide antibody and application

Monoclonal antibodies were created against an extended portion of the protein which also includes the VSAQQVQGVHAR peptide using a mammalian expression system (GenScript, NJ, USA). A slot blot apparatus was used to transfer 2 µl of human serum onto nitrocellulose membrane. Synthetic peptide standard curves were generated using 16–500 pmol of peptide on the same membrane. Membranes were blocked overnight at 4 °C (10% Milk powder, PBS), followed by incubation with 1:500 dilution of mouse anti-SPP24 sera overnight at 4 °C. The membranes were washed prior to incubation with goat anti-mouse conjugated with HRP for 1 h, washed in chemiluminescence buffer, and immersed in SuperSignal West Femto (Thermofisher) working solution for 5 min. Images were then captured using the LAS4000 system for chemiluminescence detection (GE Healthcare). ImageJ (v1.52p) was used to quantify the samples^47^.

### Ethics

All samples were collected with local and institutional approvals in place. This study was approved by the Sydney South Western Area Health Services Human Research Ethics Committee (Approval code: 14/327). Eligible patients were recruited into the study after consenting. Clinical data and materials obtained for this study have also been sampled to address a variety of biological questions within our broader research group and details of these studies are acknowledged. All research was performed in accordance with the Helsinki Guidelines.

## Supplementary information

Supplementary information.

## Abbreviations

MRM: Multiple reaction monitoring
MS: Mass spectrometry
ESI: Electrospray Ionization
DDA: Data dependent acquisition
m/z: Mass‐to charge ratio
rT: Retention time
CLE: Confocal laser endomicroscopy
CLS: Confocal Laser Score
SPP24: Secreted phosphoprotein 24
IBS: Irritable bowel syndrome
IBD: Inflammatory bowel disease
EmPAI: Exponentially modified protein abundance index
