# Supplementary material for: Spp24 is associated with endocytic signalling, lipid metabolism, and discrimination of tissue integrity for ‘leaky-gut’ in inflammatory bowel disease
Source: Sci Rep. 2020 Jul 31;10:12932. doi: 10.1038/s41598-020-69746-w (PMC7395150; doi:10.1038/s41598-020-69746-w)

Supplementary data

SPP24 is associated with endocytic signalling, lipid metabolism, and discrimination of tissue integrity for 'leaky-gut' in Inflammatory Bowel Disease.

Dr Valerie C. Wasinger[1,2] (PhD); Mr Kenneth Lu[2] (Hons); Dr Yunki Yau[2,4] (PhD); Dr Justin Nash[1] (PhD); Ms Jess Lee[2] (Hons); Dr Jeff Chang[4] (PhD, MBBS); Dr Sudarshan Paramsothy[3] (MRCP,FRACP, PhD); Dr Nadeem O. Kaakoush[2] (PhD); Prof Hazel M. Mitchell[4] (PhD); Prof Rupert W. L. Leong[2,3,5] (MBBS, MD, FRACP, AGAF)

1. Bioanalytical Mass Spectrometry Facility, Mark Wainwright Analytical centre, The University of New South Wales, Australia.
2. School of Medical Sciences, The University of New South Wales, Sydney, NSW, Australia.
3. Gastroenterology Department, Concord Repatriation General Hospital, Hospital Rd, Concord, NSW, Australia.
4. School of Biotechnology and Biomolecular Sciences, The University of New South Wales, Sydney, NSW, Australia
5. Department of Gastroenterology, Bankstown-Lidcombe Hospital, Eldridge Rd, Bankstown, NSW, Australia.

Table 1: A) Summary of Confocal Leak scored high and low permeability patients analysis using pathway enrichment analysis. B) Functional pathway analysis of SPP24 serum identified binding partners highlight an association with gastrointestinal disease, signalling networks and tissue injury. The top networks associated with the identified proteins include Cancer, Gastrointestinal Disease, Lipid metabolism, and Connective tissue development pathways.

A

**Top Canonical Pathways**		
**Name**	**p-Value**	**Overlap**
LXR/RXR Activation	8.77E-67	42.1% 51/121
FXR/RXR Activation	3.44E-60	38.1% 48/126
Acute Phase Response Signaling	1.94E-54	28.8% 49/170
Complement System	1.47E-33	59.5% 22/37
Coagulation System	3.37E-32	69.0% 21/35

**Top Networks**		
**ID**	**Associated Network Functions**	**Score**
1	Lipid Metabolism, Small Molecule Biochemistry, Vitamin and Mineral Metabolism	43
2	Lipid Metabolism, Molecular Transport, Small Molecule Biochemistry	40
3	Organismal Injury and Abnormalities, Hematological System Development and Function	38
4	Cellular Movement, Immune Cell Trafficking	38
5	Humoral Immune Response, Inflammatory Response, Developmental Disorder	36

B

Diseases and Disorders	P-value range	Number of Molecules
Gastrointestinal Disease	2.32E-03 – 4.32E-10	22
Organismal Injury and Abnormalities	2.56E-3 – 4.32E-10	32
**Molecular and Cellular Functions**		
Cell-to-cell signalling and Interaction	2.70E-03 – 1.50E-07	13
Cellular Movement	2.36E-03 – 4.72E-07	13
Small Molecule Biochemistry	2.52E-03 – 4.08E-07	14
Lipid Metabolism	2.52E-03 – 1.89E-06	12
**Physiological System Function**		
Tissue Morphology	2.32E-03 – 4.08E-07	15
Immune Cell Trafficking	2.36E-03 – 4.72E-07	12

Table 2: Primary binding partners of SPP24 peptide (FDR<1%).

Accession	Score	Mass	# significant matches	# significant sequences	EmPAI	Description
APOB_HUMAN	28700	515283	1035	534	34.06	Apolipoprotein B-100 OS=Homo sapiens GN=APOB PE=1 SV=2
CO3_HUMAN	10658	187030	391	176	22.59	Complement C3 OS=Homo sapiens GN=C3 PE=1 SV=2
APOA1_HUMAN	4186	30759	164	48	137.39	Apolipoprotein A-I OS=Homo sapiens GN=APOA1 PE=1 SV=1
ITIH2_HUMAN	2977	106397	124	70	7.41	Inter-alpha-trypsin inhibitor heavy chain H2 OS=Homo sapiens GN=ITIH2 PE=1 SV=2
SPP24_HUMAN	2240	24322	118	11	2.81	Secreted phosphoprotein 24 OS=Homo sapiens GN=SPP2 PE=1 SV=1
CO4A_HUMAN	2837	192664	118	82	2.81	Complement C4-A OS=Homo sapiens GN=C4A PE=1 SV=2
CO4B_HUMAN	2868	192631	117	80	2.75	Complement C4-B OS=Homo sapiens GN=C4B PE=1 SV=2
ITIH1_HUMAN	3677	101326	108	63	7.31	Inter-alpha-trypsin inhibitor heavy chain H1 OS=Homo sapiens GN=ITIH1 PE=1 SV=3
A1AT_HUMAN	2147	46707	100	59	67.84	Alpha-1-antitrypsin OS=Homo sapiens GN=SERPINA1 PE=1 SV=3

ITIH4_HUMAN	2388	103293	93	68	7.21	Inter-alpha-trypsin inhibitor heavy chain H4 OS=Homo sapiens GN=ITIH4 PE=1 SV=4
GELS_HUMAN	2469	85644	87	53	6.99	Gelsolin OS=Homo sapiens GN=GSN PE=1 SV=1
CO5_HUMAN	1581	188186	71	55	1.51	Complement C5 OS=Homo sapiens GN=C5 PE=1 SV=4
FIBA_HUMAN	1663	94914	68	39	3.61	Fibrinogen alpha chain OS=Homo sapiens GN=FGA PE=1 SV=2
CERU_HUMAN	1761	122128	68	52	2.81	Ceruloplasmin OS=Homo sapiens GN=CP PE=1 SV=1
HEP2_HUMAN	1270	57034	65	37	6.8	Heparin cofactor 2 OS=Homo sapiens GN=SERPIND1 PE=1 SV=3
ALBU_HUMAN	1238	69321	59	32	3.19	Serum albumin OS=Homo sapiens GN=ALB PE=1 SV=2
APOE_HUMAN	1630	36132	58	30	15.49	Apolipoprotein E OS=Homo sapiens GN=APOE PE=1 SV=1
FIBB_HUMAN	1429	55892	49	31	5.55	Fibrinogen beta chain OS=Homo sapiens GN=FGB PE=1 SV=2
CLUS_HUMAN	1261	52461	48	27	4.25	Clusterin OS=Homo sapiens GN=CLU PE=1 SV=1
PON1_HUMAN	1226	39706	46	25	6.06	Serum paraoxonase/arylesterase 1 OS=Homo sapiens GN=PON1 PE=1 SV=3
CO8B_HUMAN	1060	67003	42	26	2.68	Complement component C8 beta chain OS=Homo sapiens GN=C8B PE=1 SV=3
A2MG_HUMAN	1065	163188	41	34	0.92	Alpha-2-macroglobulin OS=Homo sapiens GN=A2M PE=1 SV=3
KAIN_HUMAN	876	48511	34	27	4.64	Kallistatin OS=Homo sapiens GN=SERPINA4 PE=1 SV=3
PROS_HUMAN	676	75074	34	25	1.84	Vitamin K-dependent protein S OS=Homo sapiens GN=PROS1 PE=1 SV=1
THRB_HUMAN	717	69992	34	20	1.58	Prothrombin OS=Homo sapiens GN=F2 PE=1 SV=2
FIBG_HUMAN	881	51479	33	25	3.55	Fibrinogen gamma chain OS=Homo sapiens GN=FGG PE=1 SV=3
CO9_HUMAN	863	63133	32	25	2.29	Complement component C9 OS=Homo sapiens GN=C9 PE=1 SV=2

K2C1_HUMAN	698	65999	31	20	1.73	Keratin, type II cytoskeletal 1 OS=Homo sapiens GN=KRT1 PE=1 SV=6
IGHG1_HUMAN	500	36083	29	13	1.93	Ig gamma-1 chain C region OS=Homo sapiens GN=IGHG1 PE=1 SV=1
C1R_HUMAN	640	80067	28	19	1.21	Complement C1r subcomponent OS=Homo sapiens GN=C1R PE=1 SV=2
C1S_HUMAN	758	76635	26	19	1.2	Complement C1s subcomponent OS=Homo sapiens GN=C1S PE=1 SV=1
APOL1_HUMAN	674	43947	25	16	2.41	Apolipoprotein L1 OS=Homo sapiens GN=APOL1 PE=1 SV=5
TTHY_HUMAN	839	15877	25	17	21.53	Transthyretin OS=Homo sapiens GN=TTR PE=1 SV=1
ANGT_HUMAN	829	53121	25	19	1.92	Angiotensinogen OS=Homo sapiens GN=AGT PE=1 SV=1
IGHG2_HUMAN	412	35878	24	8	1.11	Ig gamma-2 chain C region OS=Homo sapiens GN=IGHG2 PE=1 SV=2
PGRP2_HUMAN	689	62178	23	15	1.07	N-acetylmuramoyl-L-alanine amidase OS=Homo sapiens GN=PGLYRP2 PE=1 SV=1
ALS_HUMAN	542	65994	22	19	1.38	Insulin-like growth factor-binding protein complex acid labile subunit OS=Homo sapiens GN=IGFALS PE=1 SV=1
CNDP1_HUMAN	798	56670	22	15	1.21	Beta-Ala-His dipeptidase OS=Homo sapiens GN=CNDP1 PE=1 SV=4
PHLD_HUMAN	505	92278	22	19	0.99	Phosphatidylinositol-glycan-specific phospholipase D OS=Homo sapiens GN=GPLD1 PE=1 SV=3
K1C10_HUMAN	847	58792	21	18	1.78	Keratin, type I cytoskeletal 10 OS=Homo sapiens GN=KRT10 PE=1 SV=6
IGKC_HUMAN	581	11602	21	4	1.67	Ig kappa chain C region OS=Homo sapiens GN=IGKC PE=1 SV=1
TSP1_HUMAN	612	129300	21	17	0.49	Thrombospondin-1 OS=Homo sapiens GN=THBS1 PE=1 SV=2
LAC2_HUMAN	484	11287	20	6	3.55	Ig lambda-2 chain C regions OS=Homo sapiens GN=IGLC2 PE=1 SV=1
K1C9_HUMAN	815	62027	20	15	1.39	Keratin, type I cytoskeletal 9 OS=Homo sapiens GN=KRT9 PE=1 SV=3
CPN2_HUMAN	355	60518	20	17	1.33	Carboxypeptidase N subunit 2 OS=Homo sapiens GN=CPN2 PE=1 SV=3

VTNC_HUMAN	509	54271	20	8	0.65	Vitronectin OS=Homo sapiens GN=VTN PE=1 SV=1
APOA4_HUMAN	388	45371	19	17	2.07	Apolipoprotein A-IV OS=Homo sapiens GN=APOA4 PE=1 SV=3
FA5_HUMAN	496	251546	19	18	0.24	Coagulation factor V OS=Homo sapiens GN=F5 PE=1 SV=4
PLTP_HUMAN	611	54705	18	13	1.28	Phospholipid transfer protein OS=Homo sapiens GN=PLTP PE=1 SV=1
IGHG4_HUMAN	339	35918	18	9	1.11	Ig gamma-4 chain C region OS=Homo sapiens GN=IGHG4 PE=1 SV=1
FBLN1_HUMAN	494	77162	18	13	0.8	Fibulin-1 OS=Homo sapiens GN=FBLN1 PE=1 SV=4
ITIH3_HUMAN	377	99787	18	16	0.67	Inter-alpha-trypsin inhibitor heavy chain H3 OS=Homo sapiens GN=ITIH3 PE=1 SV=2
K22E_HUMAN	491	65393	17	16	1.09	Keratin, type II cytoskeletal 2 epidermal OS=Homo sapiens GN=KRT2 PE=1 SV=2
FETUA_HUMAN	404	39300	16	9	1.31	Alpha-2-HS-glycoprotein OS=Homo sapiens GN=AHSG PE=1 SV=1
HBB_HUMAN	498	15988	16	9	5.17	Hemoglobin subunit beta OS=Homo sapiens GN=HBB PE=1 SV=2
CO8G_HUMAN	348	22264	16	10	3.27	Complement component C8 gamma chain OS=Homo sapiens GN=C8G PE=1 SV=3
AACT_HUMAN	497	47621	16	15	1.57	Alpha-1-antichymotrypsin OS=Homo sapiens GN=SERPINA3 PE=1 SV=2
IGLL5_HUMAN	287	23049	16	6	1.15	Immunoglobulin lambda-like polypeptide 5 OS=Homo sapiens GN=IGLL5 PE=2 SV=2
CBPB2_HUMAN	371	48393	16	11	1.1	Carboxypeptidase B2 OS=Homo sapiens GN=CPB2 PE=1 SV=2
HRG_HUMAN	416	59541	16	11	0.74	Histidine-rich glycoprotein OS=Homo sapiens GN=HRG PE=1 SV=1
HPTR_HUMAN	393	39005	15	13	1.71	Haptoglobin-related protein OS=Homo sapiens GN=HPR PE=2 SV=2
ZPI_HUMAN	291	50674	15	12	1.03	Protein Z-dependent protease inhibitor OS=Homo sapiens GN=SERPINA10 PE=1 SV=1
IGHA1_HUMAN	493	37631	14	9	1.04	Ig alpha-1 chain C region OS=Homo sapiens GN=IGHA1 PE=1 SV=2

PCYOX_HUMAN	449	56604	14	13	0.99	Prenylcysteine oxidase 1 OS=Homo sapiens GN=PCYOX1 PE=1 SV=3
IGHM_HUMAN	348	49276	14	8	0.73	Ig mu chain C region OS=Homo sapiens GN=IGHM PE=1 SV=3
PON3_HUMAN	255	39582	13	12	1.47	Serum paraoxonase/lactonase 3 OS=Homo sapiens GN=PON3 PE=1 SV=3
CETP_HUMAN	391	54721	13	11	0.93	Cholesteryl ester transfer protein OS=Homo sapiens GN=CETP PE=1 SV=2
K1C16_HUMAN	395	51236	13	12	1.14	Keratin, type I cytoskeletal 16 OS=Homo sapiens GN=KRT16 PE=1 SV=4
IC1_HUMAN	433	55119	13	10	0.82	Plasma protease C1 inhibitor OS=Homo sapiens GN=SERPING1 PE=1 SV=2
A2AP_HUMAN	429	54531	13	10	0.73	Alpha-2-antiplasmin OS=Homo sapiens GN=SERPINF2 PE=1 SV=3
TFR1_HUMAN	346	84818	13	13	0.59	Transferrin receptor protein 1 OS=Homo sapiens GN=TFRC PE=1 SV=2
VWF_HUMAN	378	309058	13	12	0.13	von Willebrand factor OS=Homo sapiens GN=VWF PE=1 SV=4
CBPN_HUMAN	312	52253	12	9	0.88	Carboxypeptidase N catalytic chain OS=Homo sapiens GN=CPN1 PE=1 SV=1
CO7_HUMAN	282	93457	12	10	0.38	Complement component C7 OS=Homo sapiens GN=C7 PE=1 SV=2
FINC_HUMAN	484	262460	12	12	0.15	Fibronectin OS=Homo sapiens GN=FN1 PE=1 SV=4
APOA2_HUMAN	192	11168	11	8	6.67	Apolipoprotein A-II OS=Homo sapiens GN=APOA2 PE=1 SV=1
LBP_HUMAN	444	53350	11	7	0.48	Lipopolysaccharide-binding protein OS=Homo sapiens GN=LBP PE=1 SV=3
SAA4_HUMAN	195	14737	11	7	4.85	Serum amyloid A-4 protein OS=Homo sapiens GN=SAA4 PE=1 SV=2
ANT3_HUMAN	259	52569	11	10	0.77	Antithrombin-III OS=Homo sapiens GN=SERPINC1 PE=1 SV=1
CO8A_HUMAN	280	65121	11	7	0.59	Complement component C8 alpha chain OS=Homo sapiens GN=C8A PE=1 SV=2
C4BPA_HUMAN	294	66989	11	8	0.5	C4b-binding protein alpha chain OS=Homo sapiens GN=C4BPA PE=1 SV=2

CD14_HUMAN	267	40051	10	7	0.69	Monocyte differentiation antigen CD14 OS=Homo sapiens GN=CD14 PE=1 SV=2
IPSP_HUMAN	309	45646	10	10	0.93	Plasma serine protease inhibitor OS=Homo sapiens GN=SERPINA5 PE=1 SV=3
SHBG_HUMAN	320	43752	10	9	0.85	Sex hormone-binding globulin OS=Homo sapiens GN=SHBG PE=1 SV=2
HPT_HUMAN	266	45177	10	9	0.82	Haptoglobin OS=Homo sapiens GN=HP PE=1 SV=1
K1C14_HUMAN	217	51529	10	9	0.79	Keratin, type I cytoskeletal 14 OS=Homo sapiens GN=KRT14 PE=1 SV=4
IGHA2_HUMAN	323	36503	10	7	0.77	Ig alpha-2 chain C region OS=Homo sapiens GN=IGHA2 PE=1 SV=3
LCAT_HUMAN	349	49546	10	8	0.72	Phosphatidylcholine-sterol acyltransferase OS=Homo sapiens GN=LCAT PE=1 SV=1
K2C6C_HUMAN	262	59988	10	10	0.65	Keratin, type II cytoskeletal 6C OS=Homo sapiens GN=KRT6C PE=1 SV=3
K2C5_HUMAN	254	62340	10	10	0.62	Keratin, type II cytoskeletal 5 OS=Homo sapiens GN=KRT5 PE=1 SV=3
PLSL_HUMAN	170	70244	10	10	0.54	Plastin-2 OS=Homo sapiens GN=LCP1 PE=1 SV=6
PZP_HUMAN	187	163760	10	10	0.2	Pregnancy zone protein OS=Homo sapiens GN=PZP PE=1 SV=4
APOA_HUMAN	254	500995	10	9	0.06	Apolipoprotein(a) OS=Homo sapiens GN=LPA PE=1 SV=1
ACTB_HUMAN	304	41710	9	8	0.77	Actin, cytoplasmic 1 OS=Homo sapiens GN=ACTB PE=1 SV=1
APMAP_HUMAN	169	46451	9	9	0.79	Adipocyte plasma membrane-associated protein OS=Homo sapiens GN=APMAP PE=1 SV=2
FA9_HUMAN	202	51745	9	9	0.69	Coagulation factor IX OS=Homo sapiens GN=F9 PE=1 SV=2
QSOX1_HUMAN	341	82526	9	7	0.29	Sulfhydryl oxidase 1 OS=Homo sapiens GN=QSOX1 PE=1 SV=3
APOD_HUMAN	90	21262	8	3	0.51	Apolipoprotein D OS=Homo sapiens GN=APOD PE=1 SV=1
HBA_HUMAN	201	15248	8	5	1.6	Hemoglobin subunit alpha OS=Homo sapiens GN=HBA1 PE=1 SV=2

CBG_HUMAN	201	45112	8	7	0.59	Corticosteroid-binding globulin OS=Homo sapiens GN=SERPINA6 PE=1 SV=1
KNG1_HUMAN	187	71912	8	8	0.4	Kininogen-1 OS=Homo sapiens GN=KNG1 PE=1 SV=2
PLMN_HUMAN	232	90510	8	7	0.26	Plasminogen OS=Homo sapiens GN=PLG PE=1 SV=2
APOM_HUMAN	132	21239	7	6	1.64	Apolipoprotein M OS=Homo sapiens GN=APOM PE=1 SV=2
FETUB_HUMAN	143	42028	7	6	0.53	Fetuin-B OS=Homo sapiens GN=FETUB PE=1 SV=2
FBLN3_HUMAN	205	54604	7	5	0.32	EGF-containing fibulin-like extracellular matrix protein 1 OS=Homo sapiens GN=EFEMP1 PE=1 SV=2
PRG4_HUMAN	248	150984	7	7	0.15	Proteoglycan 4 OS=Homo sapiens GN=PRG4 PE=1 SV=2
HBD_HUMAN	186	16045	7	5	1.47	Hemoglobin subunit delta OS=Homo sapiens GN=HBD PE=1 SV=2
CRAC1_HUMAN	164	71376	7	7	0.34	Cartilage acidic protein 1 OS=Homo sapiens GN=CRTAC1 PE=1 SV=2
MA1A1_HUMAN	149	72922	7	7	0.34	Mannosyl-oligosaccharide 1,2-alpha-mannosidase IA OS=Homo sapiens GN=MAN1A1 PE=1 SV=3
PROC_HUMAN	193	52037	6	5	0.33	Vitamin K-dependent protein C OS=Homo sapiens GN=PROC PE=1 SV=1
PROZ_HUMAN	124	44715	6	4	0.31	Vitamin K-dependent protein Z OS=Homo sapiens GN=PROZ PE=1 SV=2
CO2_HUMAN	206	83214	6	6	0.24	Complement C2 OS=Homo sapiens GN=C2 PE=1 SV=2
APOC3_HUMAN	220	10846	5	4	2.71	Apolipoprotein C-III OS=Homo sapiens GN=APOC3 PE=1 SV=1
CADH5_HUMAN	119	87474	5	5	0.19	Cadherin-5 OS=Homo sapiens GN=CDH5 PE=1 SV=5
G3P_HUMAN	235	36030	5	5	0.51	Glyceraldehyde-3-phosphate dehydrogenase OS=Homo sapiens GN=GAPDH PE=1 SV=3
PAFA_HUMAN	145	50045	5	5	0.35	Platelet-activating factor acetylhydrolase OS=Homo sapiens GN=PLA2G7 PE=1 SV=1
FA10_HUMAN	99	54697	5	4	0.32	Coagulation factor X OS=Homo sapiens GN=F10 PE=1 SV=2

TRFE_HUMAN	121	77014	5	5	0.22	Serotransferrin OS=Homo sapiens GN=TF PE=1 SV=3
COMP_HUMAN	118	82808	5	5	0.2	Cartilage oligomeric matrix protein OS=Homo sapiens GN=COMP PE=1 SV=2
MASP2_HUMAN	218	75654	5	3	0.13	Mannan-binding lectin serine protease 2 OS=Homo sapiens GN=MASP2 PE=1 SV=4
APOC1_HUMAN	92	9326	4	3	1.48	Apolipoprotein C-I OS=Homo sapiens GN=APOC1 PE=1 SV=1
APOC2_HUMAN	223	11277	4	2	1.13	Apolipoprotein C-II OS=Homo sapiens GN=APOC2 PE=1 SV=1
KV106_HUMAN	142	11781	4	2	1.08	Ig kappa chain V-I region EU OS=Homo sapiens PE=1 SV=1
KV117_HUMAN	126	11757	4	2	1.08	Ig kappa chain V-I region Scw OS=Homo sapiens PE=1 SV=1
KV102_HUMAN	131	11932	4	2	1.06	Ig kappa chain V-I region AU OS=Homo sapiens PE=1 SV=1
KV118_HUMAN	130	11833	4	2	1.06	Ig kappa chain V-I region WEA OS=Homo sapiens PE=1 SV=1
HV304_HUMAN	191	12348	4	2	0.59	Ig heavy chain V-III region TIL OS=Homo sapiens PE=1 SV=1
GPX3_HUMAN	88	25537	4	4	0.59	Glutathione peroxidase 3 OS=Homo sapiens GN=GPX3 PE=1 SV=2
COL11_HUMAN	145	28647	4	4	0.51	Collectin-11 OS=Homo sapiens GN=COLEC11 PE=1 SV=1
PEDF_HUMAN	83	46283	4	4	0.3	Pigment epithelium-derived factor OS=Homo sapiens GN=SERPINF1 PE=1 SV=4
HABP2_HUMAN	70	62630	4	4	0.21	Hyaluronan-binding protein 2 OS=Homo sapiens GN=HABP2 PE=1 SV=1
C1RL_HUMAN	135	53464	4	3	0.18	Complement C1r subcomponent-like protein OS=Homo sapiens GN=C1RL PE=1 SV=2
MASP1_HUMAN	78	79195	4	4	0.16	Mannan-binding lectin serine protease 1 OS=Homo sapiens GN=MASP1 PE=1 SV=3
CFAB_HUMAN	169	85479	4	4	0.15	Complement factor B OS=Homo sapiens GN=CFB PE=1 SV=2
TMC4_HUMAN	48	79157	4	1	0.04	Transmembrane channel-like protein 4 OS=Homo sapiens GN=TMC4 PE=2 SV=3

SAA1_HUMAN	86	13524	3	3	0.9	Serum amyloid A-1 protein OS=Homo sapiens GN=SAA1 PE=1 SV=1
PRDX2_HUMAN	65	21878	3	3	0.5	Peroxiredoxin-2 OS=Homo sapiens GN=PRDX2 PE=1 SV=5
THBG_HUMAN	68	46295	3	2	0.14	Thyroxine-binding globulin OS=Homo sapiens GN=SERPINA7 PE=1 SV=2
APOC4_HUMAN	57	14543	2	2	0.49	Apolipoprotein C-IV OS=Homo sapiens GN=APOC4 PE=1 SV=1
APOF_HUMAN	100	35377	2	2	0.18	Apolipoprotein F OS=Homo sapiens GN=APOF PE=1 SV=2
AMBP_HUMAN	83	38974	2	2	0.17	Protein AMBP OS=Homo sapiens GN=AMBP PE=1 SV=1
APOA5_HUMAN	58	41187	2	2	0.16	Apolipoprotein A-V OS=Homo sapiens GN=APOA5 PE=1 SV=1
DSG2_HUMAN	52	122218	2	2	0.05	Desmoglein-2 OS=Homo sapiens GN=DSG2 PE=1 SV=2
RHG06_HUMAN	44	105882	2	1	0.03	Rho GTPase-activating protein 6 OS=Homo sapiens GN=ARHGAP6 PE=1 SV=3
LV106_HUMAN	83	11718	2	2	0.63	Ig lambda chain V-I region WAH OS=Homo sapiens PE=1 SV=1
KV302_HUMAN	140	11768	2	2	0.63	Ig kappa chain V-III region SIE OS=Homo sapiens PE=1 SV=1
LV302_HUMAN	85	11928	2	2	0.62	Ig lambda chain V-III region LOI OS=Homo sapiens PE=1 SV=1
HV303_HUMAN	124	12574	2	2	0.58	Ig heavy chain V-III region 23 OS=Homo sapiens GN=IGHV3-23 PE=1 SV=2
HV320_HUMAN	60	12722	2	2	0.57	Ig heavy chain V-III region GAL OS=Homo sapiens PE=1 SV=1
HV307_HUMAN	80	13659	2	2	0.53	Ig heavy chain V-III region CAM OS=Homo sapiens PE=1 SV=1
HBE_HUMAN	35	16192	2	2	0.43	Hemoglobin subunit epsilon OS=Homo sapiens GN=HBE1 PE=1 SV=2
KV121_HUMAN	75	12238	2	1	0.26	Ig kappa chain V-I region Ni OS=Homo sapiens PE=1 SV=1
C1QC_HUMAN	94	25757	2	2	0.26	Complement C1q subcomponent subunit C OS=Homo sapiens GN=C1QC PE=1 SV=3

TPM3_HUMAN	62	32930	2	2	0.2	Tropomyosin alpha-3 chain OS=Homo sapiens GN=TPM3 PE=1 SV=2
LUM_HUMAN	90	38405	2	2	0.17	Lumican OS=Homo sapiens GN=LUM PE=1 SV=2
1A01_HUMAN	94	40820	2	2	0.16	HLA class I histocompatibility antigen, A-1 alpha chain OS=Homo sapiens GN=HLA-A PE=1 SV=1
RUVB1_HUMAN	31	50196	2	2	0.13	RuvB-like 1 OS=Homo sapiens GN=RUVBL1 PE=1 SV=1
A1BG_HUMAN	93	54220	2	2	0.12	Alpha-1B-glycoprotein OS=Homo sapiens GN=A1BG PE=1 SV=4
GPV_HUMAN	62	60921	2	2	0.1	Platelet glycoprotein V OS=Homo sapiens GN=GP5 PE=1 SV=1
PCSK9_HUMAN	53	74239	2	2	0.08	Proprotein convertase subtilisin/kexin type 9 OS=Homo sapiens GN=PCSK9 PE=1 SV=3
F13A_HUMAN	32	83215	2	2	0.08	Coagulation factor XIII A chain OS=Homo sapiens GN=F13A1 PE=1 SV=4
TAXB1_HUMAN	46	90820	2	2	0.07	Tax1-binding protein 1 OS=Homo sapiens GN=TAX1BP1 PE=1 SV=2
F171B_HUMAN	39	92124	2	2	0.07	Protein FAM171B OS=Homo sapiens GN=FAM171B PE=2 SV=3
MUSK_HUMAN	35	96993	2	2	0.06	Muscle, skeletal receptor tyrosine-protein kinase OS=Homo sapiens GN=MUSK PE=1 SV=1
ENPP2_HUMAN	62	98930	2	2	0.06	Ectonucleotide pyrophosphatase/phosphodiesterase family member 2 OS=Homo sapiens GN=ENPP2 PE=1 SV=3
CO6_HUMAN	67	104718	2	2	0.06	Complement component C6 OS=Homo sapiens GN=C6 PE=1 SV=3
INT4_HUMAN	38	108102	2	2	0.06	Integrator complex subunit 4 OS=Homo sapiens GN=INTS4 PE=1 SV=2
CFAH_HUMAN	81	139005	2	2	0.04	Complement factor H OS=Homo sapiens GN=CFH PE=1 SV=4
CR063_HUMAN	33	77181	2	1	0.04	Uncharacterized protein C18orf63 OS=Homo sapiens GN=C18orf63 PE=2 SV=2
CO6A3_HUMAN	36	343457	2	2	0.02	Collagen alpha-3(VI) chain OS=Homo sapiens GN=COL6A3 PE=1 SV=5
TRRAP_HUMAN	30	437318	2	1	0.01	Transformation/transcription domain-associated protein OS=Homo sapiens GN=TRRAP PE=1 SV=3

BGH3_HUMAN	32	74634	1	1	0.04	Transforming growth factor-beta-induced protein ig-h3 OS=Homo sapiens GN=TGFBI PE=1 SV=1

Table 3: ESI-LC-MS/MS data of identified PON1 binding partners demonstrating binding of SPP24.The number of peptides identified along with unique peptides (in brackets) are given in the 'Peptides' column.

Accession	Peptides	Score	Anova (p)	Description
CAD22_HUMAN	2 (1)	96.68	1.99E-14	Cadherin-22
PPCEL_HUMAN	1 (1)	50.34	2.22E-14	Prolyl endopeptidase-like
HPT_HUMAN	1 (1)	43.67	7.15E-09	Haptoglobin
MLTK_HUMAN	1 (1)	41.75	2.79E-06	Mitogen-activated protein kinase MLT
CO9_HUMAN	1 (1)	45.18	2.87E-10	Complement component C9
SAA1_HUMAN	2 (1)	87.44	2.60E-03	Serum amyloid A-1 protein
ZN787_HUMAN	1 (1)	53.25	1.72E-04	Zinc finger protein 787
RABL6_HUMAN	1 (1)	41.35	1.26E-05	Rab-like protein 6
LV302_HUMAN	1 (1)	70.54	5.22E-03	Ig lambda chain V-III region LOI
SAA2_HUMAN	2 (1)	84.45	3.90E-03	Serum amyloid A-2 protein
ADCY8_HUMAN	1 (1)	43.33	2.11E-04	Adenylate cyclase type 8
K2C6A_HUMAN	3 (3)	128.76	1.01E-03	Keratin, type II cytoskeletal 6A
KV309_HUMAN	1 (1)	64.01	5.78E-04	Ig kappa chain V-III region VG (Fragment)
PEX6_HUMAN	1 (1)	45.74	3.06E-04	Peroxisome assembly factor 2 SV=2
LBP_HUMAN	1 (1)	39.52	5.87E-04	Lipopolysaccharide-binding protein
KLH36_HUMAN	1 (1)	41.70	2.90E-04	Kelch-like protein 36
WDR26_HUMAN	4 (1)	182.31	9.39E-03	WD repeat-containing protein 26
CO4A2_HUMAN	1 (1)	50.52	3.27E-03	Collagen alpha-2(IV) chain
KV303_HUMAN	1 (1)	44.88	1.73E-03	Ig kappa chain V-III region NG9 (Fragment)
SNX27_HUMAN	1 (1)	45.44	9.81E-04	Sorting nexin-27
CORA1_HUMAN	1 (1)	54.02	1.49E-04	Collagen alpha-1(XXVII) chain
PP4R4_HUMAN	1 (1)	68.77	1.87E-03	Serine/threonine-protein phosphatase 4 regulatory subunit 4
RBM26_HUMAN	1 (1)	40.33	4.69E-03	RNA-binding protein 26
CR065_HUMAN	1 (1)	46.53	2.13E-03	Putative uncharacterized protein C18orf65
SRBP1_HUMAN	1 (1)	44.37	3.75E-03	Sterol regulatory element-binding protein 1
K2C5_HUMAN	2 (2)	88.53	9.57E-06	Keratin, type II cytoskeletal 5
FIBB_HUMAN	1 (1)	55.94	1.39E-04	Fibrinogen beta chain
K1C10_HUMAN	3 (2)	154.98	3.87E-04	Keratin, type I cytoskeletal 10
CQ074_HUMAN	1 (1)	42.78	8.83E-04	Uncharacterized protein C17orf74
ITIH2_HUMAN	7 (6)	317.29	1.22E-08	Inter-alpha-trypsin inhibitor heavy chain H2
ZSWM6_HUMAN	3 (2)	128.28	1.05E-08	Zinc finger SWIM domain-containing protein 6

CO5_HUMAN	3 (3)	146.42	3.77E-05	Complement C5
NOTC2_HUMAN	2 (1)	77.29	8.44E-05	Neurogenic locus notch homolog protein 2
TTHY_HUMAN	4 (4)	187.41	1.16E-06	Transthyretin
EMIL3_HUMAN	1 (1)	40.72	8.62E-03	EMILIN-3
SIX3_HUMAN	1 (1)	56.00	2.40E-04	Homeobox protein SIX3
GSX1_HUMAN	1 (1)	57.90	6.69E-05	GS homeobox 1
ALS_HUMAN	1 (1)	42.33	1.57E-03	Insulin-like growth factor-binding protein complex acid labile subunit
LUR1L_HUMAN	1 (1)	28.70	1.61E-05	Leucine rich adaptor protein 1-like
A1AT_HUMAN	15 (15)	828.47	8.71E-05	Alpha-1-antitrypsin
K2C1_HUMAN	11 (9)	581.73	6.11E-03	Keratin, type II cytoskeletal 1 SV=6
APOC4_HUMAN	1 (1)	52.90	5.00E-03	Apolipoprotein C-IV
APOBR_HUMAN	1 (1)	45.55	1.40E-05	Apolipoprotein B receptor
JIP3_HUMAN	1 (1)	42.15	1.44E-04	C-Jun-amino-terminal kinase-interacting protein 3
NCOR1_HUMAN	1 (1)	43.03	5.46E-04	Nuclear receptor corepressor 1
RENT1_HUMAN	1 (1)	50.18	1.15E-04	Regulator of nonsense transcripts 1
LCE1F_HUMAN	1 (1)	32.12	8.31E-03	Late cornified envelope protein 1F
EPC1_HUMAN	1 (1)	42.74	3.68E-04	Enhancer of polycomb homolog 1
NFAC4_HUMAN	1 (1)	49.37	1.00E-02	Nuclear factor of activated T-cells, cytoplasmic 4
MMRN1_HUMAN	1 (1)	40.03	1.65E-06	Multimerin-1
SPP24_HUMAN	1 (1)	42.49	1.00E-02	Secreted phosphoprotein 24
GLI1_HUMAN	1 (1)	42.66	5.09E-04	Zinc finger protein GLI1
PKHG2_HUMAN	1 (1)	48.13	1.17E-04	Pleckstrin homology domain-containing family G member 2
LRSM1_HUMAN	1 (1)	44.80	4.48E-05	E3 ubiquitin-protein ligase LRSAM1
CDV3_HUMAN	2 (1)	90.65	4.83E-11	Protein CDV3 homolog
APOC3_HUMAN	5 (5)	307.92	1.94E-06	Apolipoprotein C-III
ALDOA_HUMAN	1 (1)	43.69	2.05E-05	Fructose-bisphosphate aldolase A
NPHP3_HUMAN	1 (1)	39.98	1.40E-05	Nephrocystin-3
KV104_HUMAN	2 (2)	113.27	2.05E-05	Ig kappa chain V-I region CAR
SYN1_HUMAN	1 (1)	42.91	6.31E-06	Synapsin-1
APOE_HUMAN	11 (10)	617.10	1.86E-04	Apolipoprotein E
PLAK_HUMAN	1 (1)	39.27	6.94E-04	Junction plakoglobin
COPA_HUMAN	1 (1)	48.55	2.05E-03	Coatomer subunit alpha
ANGT_HUMAN	2 (2)	108.51	6.45E-05	Angiotensinogen
ACTB_HUMAN	2 (2)	90.23	8.46E-07	Actin, cytoplasmic 1

TRFE_HUMAN	1 (1)	45.42	5.39E-03	Serotransferrin
CPNS1_HUMAN	1 (1)	45.92	1.00E-02	Calpain small subunit 1
PO3F3_HUMAN	1 (1)	41.53	2.04E-05	POU domain, class 3, transcription factor 3
ITIH1_HUMAN	2 (2)	107.72	4.70E-03	Inter-alpha-trypsin inhibitor heavy chain H1
GOR_HUMAN	1 (1)	44.13	6.48E-03	Putative exonuclease GOR

Figure 1: Validation of antibody binding specificity using immunoprecipitation with immobilised antibody. The antibody of the protein portion containing the SPP24 peptide epitope was immobilised on beads to determine proteins associating with the target from serum samples. Non-specifically bound proteins were assessed in the same manner by binding an unrelated protein to the beads and subtracting common proteins. Proteins were eluted and identified using spectral counting mass spectrometry.

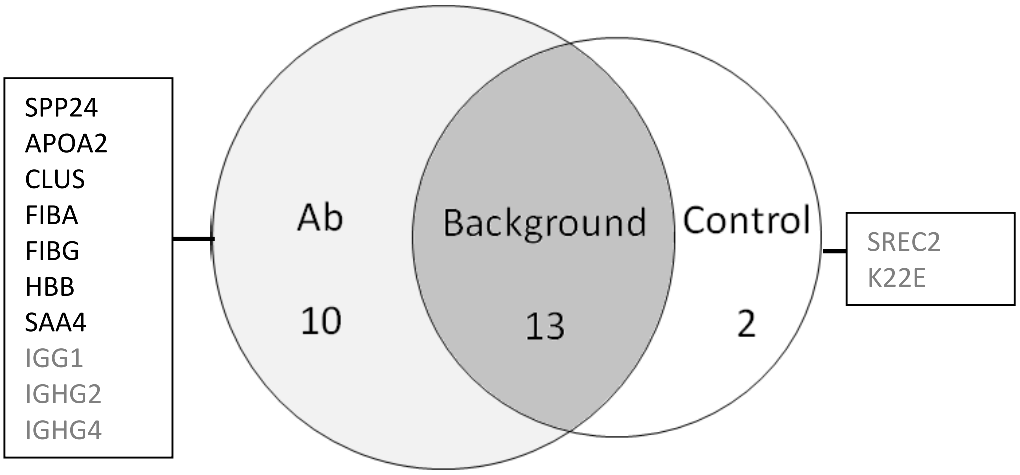

Supplement: Supplementary file 1 — Supplementary information. [file 41598_2020_69746_MOESM1_ESM.pdf]
